# Autopsy findings after long-term treatment of COVID-19 patients with microbiological correlation

**DOI:** 10.1007/s00428-020-03014-0

**Published:** 2021-01-20

**Authors:** Katja Evert, Thomas Dienemann, Christoph Brochhausen, Dirk Lunz, Matthias Lubnow, Markus Ritzka, Felix Keil, Matthias Trummer, Alexander Scheiter, Bernd Salzberger, Udo Reischl, Peter Boor, André Gessner, Jonathan Jantsch, Diego F. Calvisi, Matthias Evert, Barbara Schmidt, Michaela Simon

**Affiliations:** 1 Institute of Pathology, University of Regensburg, Franz-Josef-Strauß-Allee 11, 93053 Regensburg, Germany; 2 Department of Surgery, University Hospital Regensburg, Regensburg, Germany; 3 Department of Medicine II, University Medical Centre, Regensburg, Germany; 4 Department of Anesthesiology and Intensive Care, University Hospital Regensburg, Regensburg, Germany; 5 Department of Infection Prevention and Infectious Diseases, University Hospital Regensburg, Regensburg, Germany; 6 Institute of Clinical Microbiology and Hygiene, Regensburg University Hospital, Regensburg, Germany; 7 Institute of Pathology, University Hospital Aachen, RWTH Aachen, Aachen, Germany

**Keywords:** COVID-19, Autopsy, Fungal infection, Mycosis, Macrophage activation syndrome

## Abstract

Between April and June 2020, i.e., during the first wave of pandemic coronavirus disease 2019 (COVID-19), 55 patients underwent long-term treatment in the intensive care unit at the University Hospital of Regensburg. Most of them were transferred from smaller hospitals, often due to the need for an extracorporeal membrane oxygenation system. Autopsy was performed in 8/17 COVID-19-proven patients after long-term treatment (mean: 33.6 days). Autopsy revealed that the typical pathological changes occurring during the early stages of the disease (e.g., thrombosis, endothelitis, capillaritis) are less prevalent at this stage, while severe diffuse alveolar damage and especially coinfection with different fungal species were the most conspicuous finding. In addition, signs of macrophage activation syndrome was detected in 7 of 8 patients. Thus, fungal infections were a leading cause of death in our cohort of severely ill patients and may alter clinical management of patients, particularly in long-term periods of treatment.

**Supplementary Information:**

The online version contains supplementary material available at 10.1007/s00428-020-03014-0.

## Introduction

Clinical autopsy is an essential medical tool not only for quality assurance and education but also to improve and advance our understanding of diseases. Particularly in unknown and emerging diseases such as pandemic coronavirus disease 2019 (COVID-19), postmortem examinations are of utmost importance to gain a better understanding of the underlying pathomechanisms and disease-associated alterations in different organs. During the early phase of the COVID-19 pandemic in Germany, autopsies were not recommended due to the unknown risk of infection. After the intervention of the academic German Association of Pathologists (Deutsche Gesellschaft für Pathologie) and a subsequent public discussion, this recommendation was revoked and autopsies were performed. The first autopsy studies consistently showed similar changes of the lung and other organs, particularly consisting of neutrophilic capillaritis, microthrombosis, pulmonary thromboembolism, and signs of multi-organ failure [2] as well as diffuse alveolar damage (DAD). At later stages, squamous metaplasia as a peculiar feature and pulmonary fibrosis occur [8, 14]. Single articles discussed a higher prevalance of COVID-19-associated pulmonary aspergillosis [1], and few observations of invasive aspergillosis in patients with COVID-19 [24] have been reported. However, no systemic analysis of autopsies has been performed to document the incidence and contribution of mycotic infections during the course of the disease, particularly at later stages, to date. Here, we show that fungal infections are a constant and important finding in autopsies of COVID-19-deceased patients, which has not been detected clinically at lifetime though significantly contribute to patients deaths.

## Methods

### Autopsy

For all deceased patients, informed consent was obtained. Autopsies were performed between April and June 2020 following proposed guidelines for hazard group 3 pathogens [10]. The aerosol formation was avoided as much as possible. Lungs were fixed by perfusion with buffered formalin and dissected after a minimum of 3-day fixation. In addition, samples of all major organs were collected following standard protocols as well as other specimens that seemed to be of interest during the autopsy. All cases were reviewed by at least two experienced pathologists.

For electron microscopy, samples from the lungs, trachea, liver, heart, and kidney were taken and fixed in Karnovsky fixative. Fresh frozen samples of the latter organs underwent microbiological analyses.

### Histology and immunohistology

Routine histology stainings (hematoxylin and eosin stain (HE), periodic acid–Schiff (PAS) reaction, Prussian blue (Fe), Elastica-van-Gieson (EvG), and Azan staining) were performed following standard protocols. Immunohistology was performed in selected cases (CD 68 PGM1, type PiZ-alpha-1-antitrypsin) using an automated immunostainer (Ventana Benchmark Ultra, Roche Diagnostics, Mannheim, Germany).

### Electron microscopy

A total of 35 samples were examined by electron microscopy. For this purpose, tissue samples were fixed in 0.1 M cacodylate-buffered Karnovsky solution for at least 72 h at room temperature, followed by postfixation in 1% osmium tetroxide for 2 h. Subsequently, samples were embedded in Epon, using the Lynx^TM^ EL Microscopy tissue processor (Electron microscopy sciences, Hatfield, PA, USA). Semi-thin-sections were cut using the Reichert Ultracut S Microtome (Leica-Reichert, Wetzlar, Germany) to identify the areas of interest. Ultra-thin sections (60-nm thickness) from these regions were successively cut. Sections were transferred on ultra-sonic cleaned EM-Grids, dried, contrasted with 2%-uranyl-acetate for 10 min, and then covered with 2%-lead-citrate for 10 min. After contrasting, the slides were carefully washed with bidestilled water. Electron-microscopy was performed using the LEO 912 AB-electron-microscope (Zeiss, Oberkochen, Germany) operating at 100 kV.

### SARS-CoV-2 PCR

Nucleic acids were extracted from fresh-frozen organ slices using the EZ1 Virus Mini Kit v2.0 with the EZ1 Advanced XL system (Qiagen, Hilden, Germany). Viral (+)ssRNA was amplified using the SARS-CoV-2 E gene RT-PCR [4] with StepOnePlus Real-Time PCR System (ThermoFisherScientific, Schwerte, Germany). Bacteriophage MS2 served as an internal control for extraction and amplification efficacy [7].

### Mycological examinations

Samples were either taken antemortem or postmortem fresh-frozen (FF) or after formalin-fixation and paraffin embedding (FFPE) and subjected to PCR analysis for the detection of fungal pathogens and/or to fungal culture. In total, 95 samples were analyzed. Three samples were analyzed for patient 1 (P1; 1 respiratory secretion, 1 postmortem FF, and 1 FFPE lung tissue sample), 2 samples for P2 (1 respiratory secretion and 1 FFPE lung biopsy), 24 samples for P3 (4 respiratory secretions, 8 serum samples, 1 liver, 1 intestinal, and 1 gallbladder tissue specimen, and 9 postmortal FF samples including the liver [*n* = 2], intestinum [*n* = 1], kidney [*n* = 2], heart [*n* = 2], lung [*n* = 1], and 1 FFPE intestinal sample), 23 samples for P4 (7 respiratory secretions, 8 serum samples, 1 EDTA stabilized blood sample, 1 EDTA plasma sample, and 6 postmortal FF samples including intestinal tissue [*n* = 1], lung [*n* = 2], heart [*n* = 1], kidney [*n* = 1], and liver [*n* = 1]), 20 samples for P5 (3 respiratory secretions, 15 serum samples, and 2 postmortal samples encompassing 1 FF lung tissue sample and 1 FFPE esophageal tissue sample), 8 samples for P6 (5 respiratory secretions and 3 postmortal samples including 1 FF and 2 FFPE lung tissue samples), 13 samples for P7 (12 respiratory secretions and 1 FFPE skin ulcer sample), and 2 respiratory secretions for P8.

PCR assays for the specific detection of *Pneumocystis jirovecii, Mucorales, Aspergillus fumigatus,* and *Aspergillus* spp. DNA were performed as described previously [16, 17, 23]. For the cultivation of fungi, Sabouraud-2% Glucose-agar plates and Sabouraud-2% Glucose-broths (Merck) were inoculated with patient specimens and incubated at 35 ± 1 °C for 7 and 28 days, respectively.

## Results

### Patient characteristics

A total of 47.1% (*n* = 8) of the deceased patients with proven COVID-19-infection at the University Hospital of Regensburg underwent a full-body autopsy (4 female, 4 male). The median age was 62 years (44–73 years), and the average time of hospitalization was 33.6 days (16–56 days). All suffered from non-severe comorbidities: 7 were obese (median BMI of obese: 31.5 (26.2–40.4) kg/m^2^) and 5 had arterial hypertension. One known severe disease was liver cirrhosis in one patient (P4), clinically being attributed to chronic alcohol abuse. Autoptically, however, we were able to detect an alpha-1-antitrypsin deficiency, whose course was possibly unfavorably influenced by alcohol consumption. All patients received maximum therapy, including mechanical ventilation, dialysis, and ECMO. A summary of the patient characteristics is provided in Supplementary Table 1, while Table 1 shows the clinical, microbiological, and pathologic findings in detail.

### Main autopsy findings and fungal-related microbiological findings

#### Lung pathology and known COVID-19-related systemic findings

**Fig. 1:**
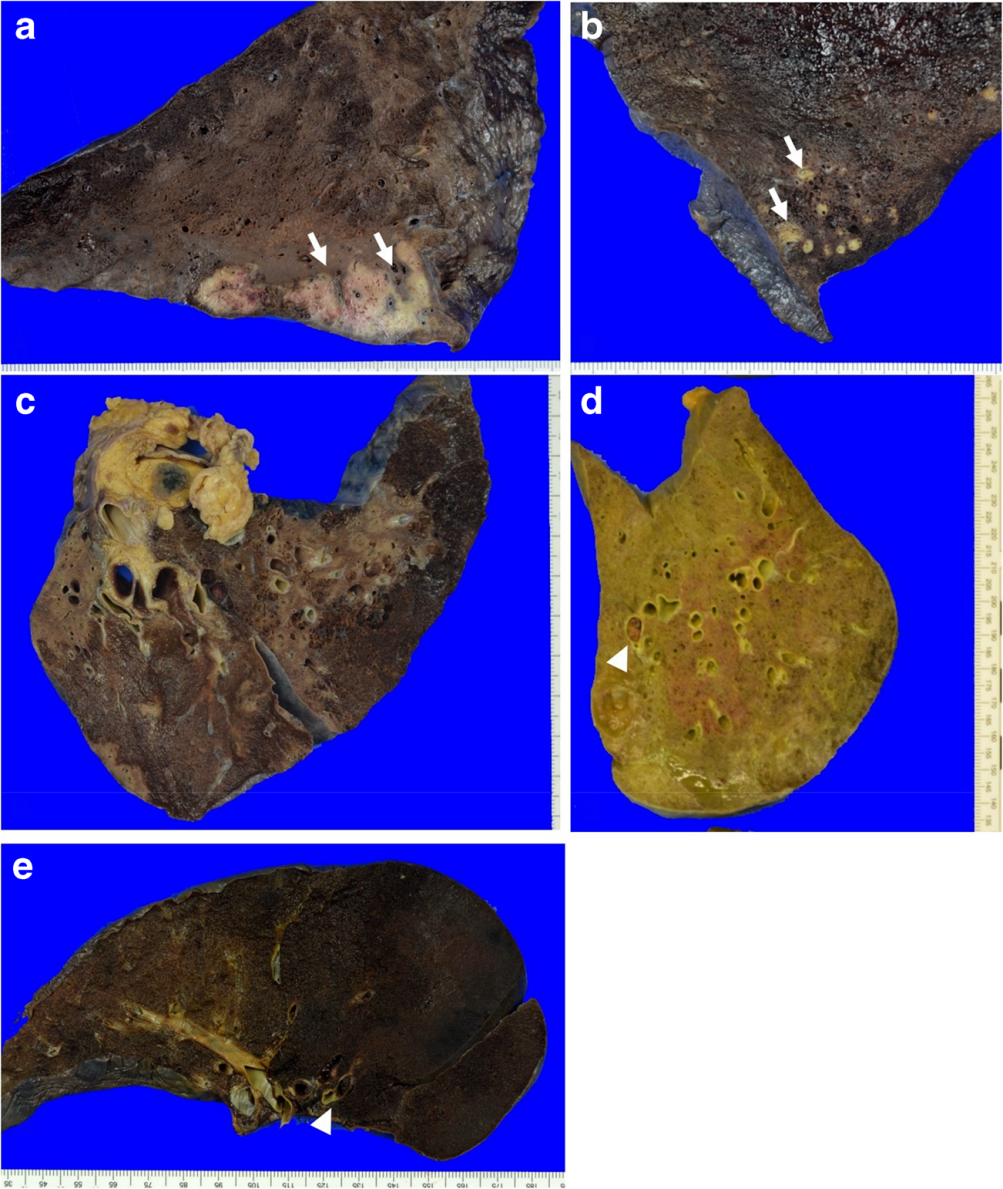
Macroscopical lung pathology: White nodules with sharply demarcated borders (**a** P1, **b** P2, arrow). In **c**, a mixture of dark red and paler brown lung parenchyma is visible (P5). Thrombi within peripheral branches of the pulmonary artery (arrowhead **d** P7, **e** P4). **d** Additional icteric changes (P7). In **e**, the thrombus is accompanied by a hemorrhagic infarction of the lung (P4)

**Fig. 2:**
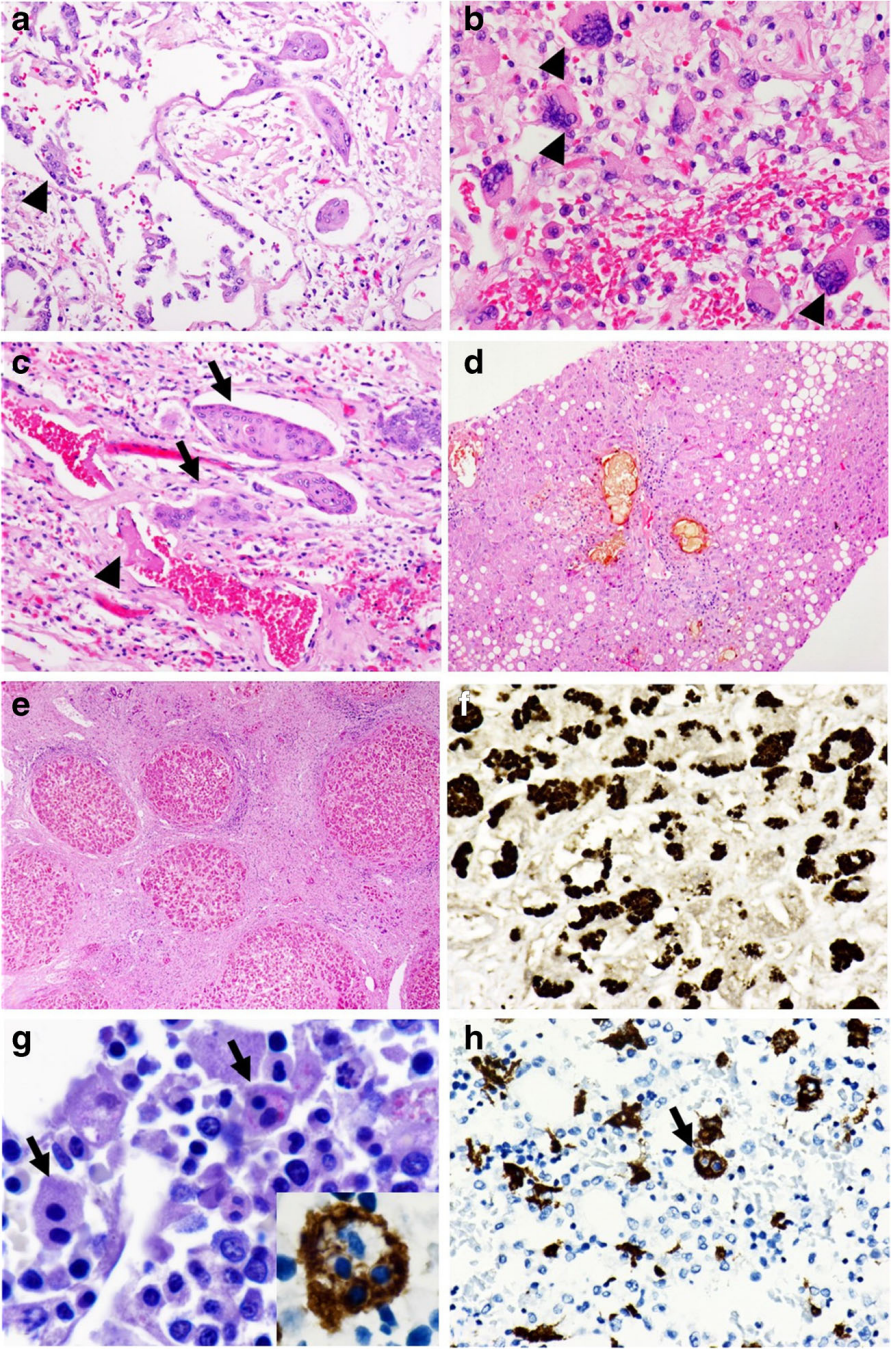
Histological findings in COVID-19-deceased: Typical changes of the lung in COVID-19 deceased with hyperplasia of type II pneumocytes (**a** P5, arrowhead, HE, original magnification × 200), accompanying giant cells (**b** P5, arrowhead, HE, original magnification × 400), and squamous metaplasia (**c** P5, arrow, HE, original magnification × 200). Next to this, a small fresh hyaline microthrombus could be found (**c**: arrowhead). Sepsis-associated hepatic injury with marked cholestasis and steatosis (**d** P3, HE, original magnification × 100). Liver cirrhosis (**e** P4, HE, original magnification × 40) with globules of alpha-1-antitrypsin (**f** P4, Alpha-1-Antitrypsin, type PIZ, original magnification × 400). Bone marrow with signs of macrophage activation syndrome (**g** P1, arrowhead, PAS, original magnification × 400; **h** P1, arrow, CD68, original magnification × 400)

**Fig. 3:**
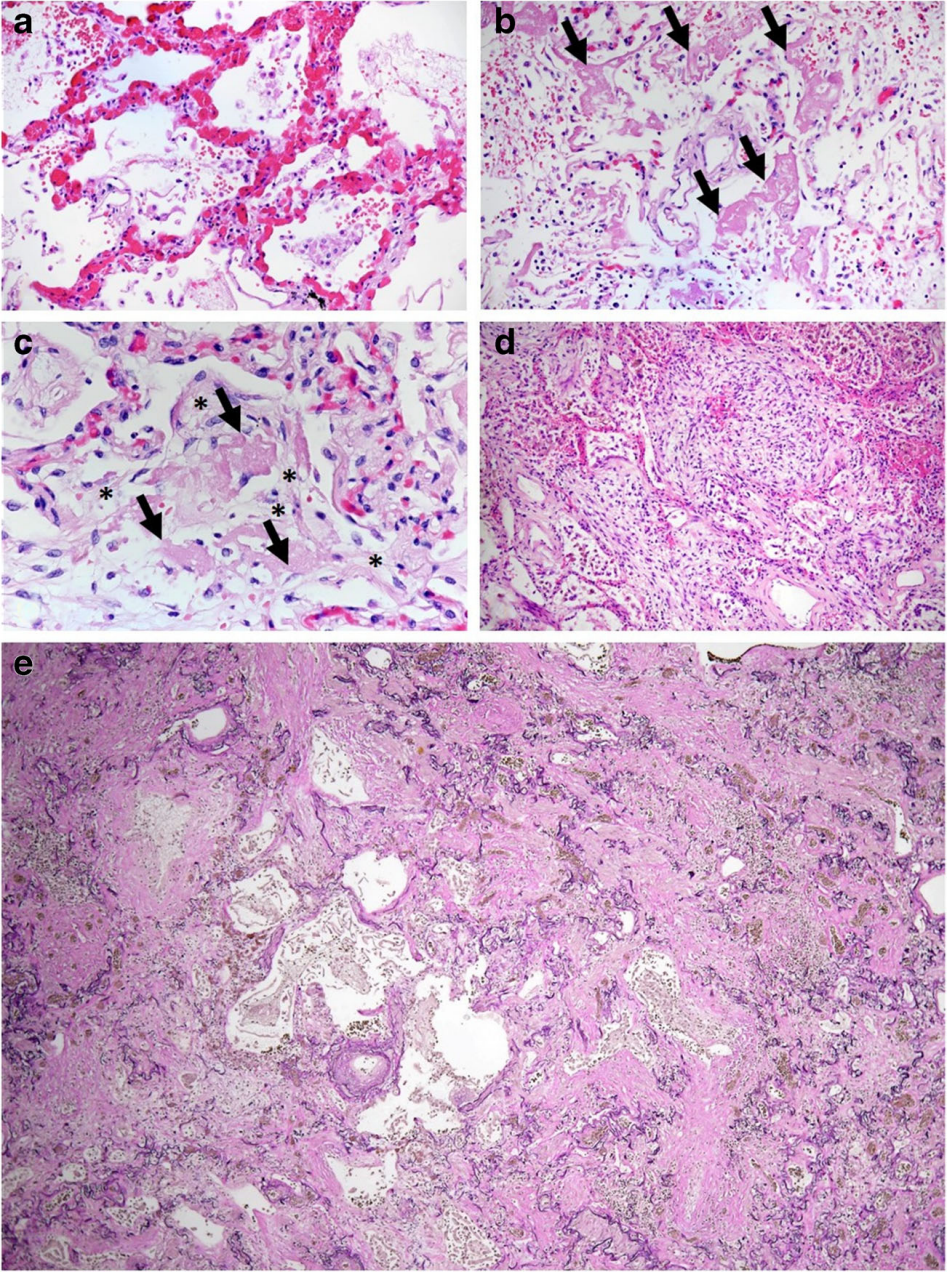
Histological examples of diffuse alveolar damage (DAD) observed in this study: Very early stages of exudative damage (acute pattern) such as alveolar cell desquamation, congestion, and loss of type 1-pneumocytes were only rarely seen (**a**). Also, typical hyaline membranes (= alveolar fibrin deposits; arrows), which develop later during the acute (exudative) pattern (**b**), could only be demonstrated occasionally. In addition, most of these hyaline membranes already showed signs of beginning fibrosis (**c**; arrows on fibrin, asterisks on organizing foci), classifying these cases as being an intermediate step (= proliferative pattern) to the fibrotic stage. Corresponding to the generally long duration of mechanical ventilation (mean 29 days), DAD in our patients often corresponded to early fibrotic stages, characterized by myofibroblast proliferation with increasing interstitial as well as alveolar collagen deposition (**d**). Only one patient (patient 8) showed a severe pulmonary fibrosis with dense collagen deposits, large areas of nearly complete loss of normal alveolar architecture and elastic fibers and microcysts as a final and fatal consequence of DAD (**e**). Respiratory failure was also the main cause of death in this case. Original magnification: × 200 (**a**, **b**), × 400 (**c**), × 100 (**d**), × 40 (**e**). Staining: HE (**a**–**d**), elastic van Gieson (**e**)

While early histological signs of COVID 19 disease such as capillaritis/vasculitis and endothelitis—albeit extensive microscopic tissue examination—have not been found in a single case in this autopsy series, thrombosis was detected in 60% of the patients, consisting of small to medium-sized thrombi in peripheral pulmonary arteries that were already found macroscopically and histologically confirmed in 5 out of 8 cases (P1, P3, P4, P5, P7; Fig. 1d, 1e). Thrombi were usually not fresh but showed advanced stages of organization. Together with the absence of venous thrombosis, this finding indicates that these thrombi were indeed remnants of COVID-19-related early-stage thrombosis and not the result of non-specific pulmonary thromboembolism. In one case, the thrombus was associated with a corresponding hemorrhagic infarction of the lung tissue (P4; Fig. 1e), but, because of the small extent of vessel occlusion in general, signs of pulmonary hypertension such as right heart congestion were not detected. Other non-specific alterations of COVID-19-infection such as type-II-pneumocytic hyperplasia and squamous metaplasia of alveolar spaces were observed in 100% and 25% of the cases, respectively (Fig. 2a–c). All but one patient (P3) showed severe DAD. Histologically, besides mycotic pneumonia, DAD was the most common lung pathology and presented usually in late exsudative and proliferative and in 5/8 cases already in fibrotic stages of the disease. A detailed description of DAD histomorphology is shown in Fig. 3. Marked iron deposits in intraalveolar macrophages were seen in 7 of 8 cases, while megakaryocyte embolisms were present in all cases.

**Table 1:** Patient characteristics, histopathological, and microbiological findings

Patient	1	2	3	4	5	6	7	8
Clinical data								
Age	68	73	44	61	52	68	66	67
Sex	f	m	f	f	m	m	m	f
Body Mass Index (kg/m^2^)	35.4	26.2	40.4	20.8	29.2	34	27.8	27.7
Comorbidities	Hypertension Obesity Depression	Hypertension Obesity COPD HLP NASH	Obesity	Liver cirrhosis	Obesity Sleep apnea syndrome Factor V deficiency	Hypertension Obesity Atrial fibrillation Diabetes	Hypertension IHD	Hypertension Obesity Atrial fibrillation
Preexisting diabetes	n	n	n	n	n	y	n	n
Days between onset of first symptoms and death	35	21	26	21	37	54	Unknown	44
Days being hospitalized	21	16	28	34	31	48	56	37
Days on mechanical ventilation	21	13	24	21	28	48	51	31
ECMO	y, VA	n	y, VV, and VA	n	y, VA	y, VV	n	y, VV
Dialysis	y, 1d	y, 9d	y, 17d	y, 12d	y, 26d	y, 41	y, 49	y, 1
Convalescent plasma treatment	n	n	y	y	y	n	y	n
Preexisting immunsuppressive therapy	n	n	n	n	n	n	n	n
Immunsuppressive therapy	Steroids (vasoplegia)	Steroids (vasoplegia)	Steroids (vasoplegia)	Steroids (vasoplegia)	Steroids/tozilizumab	Steroids/anakinra	Steroids/anakinra	Steroids (vasoplegia)
Hyperinflammatory state	y 1985 pg/ml IL-6	y 8296 pg/ml IL-6	y 2271 pg/ml IL-6	y 4427 pg/ml IL-6	y 3829 pg/ml IL-6	y 6821 pg/ml IL-6	n 264 pg/ml IL-6	y 1728 pg/ml IL-6
D-Dimer (mg/l) (hospital admission/peak/death)	4.82/8.83/6.04	4.25/8.8/0.36	3.9/6.84/6.5	5.46/> 35/> 35	2.54/4.74/1.3	2.0/29.01/19.5	0.36/1.21/1.17	12.09/14.8/14.8
Days on antibiotics	2	12	26	30	26	48	56	37
Days on antimycotics	0	1	6	17	8	7	36	1
Kinetic therapy	y	y	y	n	y	y	y	y
Main autopsy findings								
Lung:								
Endothelitis	−	−	−	−	−	−	−	−
Vasculitis	−	−	−	−	−	−	−	−
Thrombosis*	+	−	+	+	+	−	+	−
Hyperplasia of type-II-pneumocytes	+	+	+	+	+	+	+	+
Squamous cell metaplasia	−	+	−	−	+	−	−	−
Signs of diffuse alveolar damage	+	+	−	+	+	+	+	+
Liver	Mild steatosis	Mild fibrosis, mild steatosis, mild cholestasis	Moderate steatosis, moderate cholestasis	Cirrhosis, mild cholestasis	Moderate cholestasis	Mild fibrosis, mild steatosis, mild cholestasis	Mild cholestasis	Mild steatosis, mild cholestasis
Bone marrow								
Hemophagoyctosis	+	+	−	+	+	+	+	+
Hemophagocytosis diagnosis clinically possible in retrospect?	Maybe**	Maybe**	No	No	No	Yes (diagnosed before death)	Yes (diagnosed before death)	Maybe**
Fungal detection	+ (lung, angioinvasive aspergillus)	+ (lung, angioinvasive aspergillus)	+ (gut, angioinvasive mucor)	+ (lung, angioinvasive mucor)	+ (esophagus, aspergillus)	+ (lung, angioinvasive aspergillus)	+ (skin)	-
Cause of death	Septic multiorgan failure	Septic multiorgan failure	Septic multiorgan failure	Respiratory multiorgan failure	Septic multiorgan failure	Septic multiorgan failure	Septic multiorgan failure	Respiratory multiorgan failure
Microbiological findings								
SARS CoV-2 PCR								
Lung	4.3*10^3^	5.0*10^3^	6.3*10^6^	7.0*10^5^	Negative	< 3.0*10^2^	< 3.0*10^2^	Negative
Trachea	1.0*10^3^	< 3.0*10^2^	Not tested	Not tested	Not tested	Negative	Not tested	Not tested
Kidney	Negative	< 3.0*10^2^	3.5*10^2^	6.0*10^2^	Negative	Negative	Negative	Negative
Liver	Negative	3.6*10^2^	8.9*10^3^	5.0*10^4^	Negative	Negative	Negative	Negative
Heart	Negative	Negative	5.5*10^3^	1.1*10^5^	Negative	Negative	Negative	Negative
Gut	Not tested	Not tested	1.6 × 10^5^	Not tested	Not tested	Not tested	Not tested	Not tested
PCR detection of fungal pathogens	Fungal pathogens not detected	Fungal pathogens not detected	Detection of *Rhizopus microsporus*/*azygosporus* in gastro-intestinal and myocardial samples	Detection of *Rhizopus microsporus*/*azygosporus* in respiratory and myocardial samples	Detection of *Aspergillus fumigatus* and *Mucor* spp. in an esophageal ulcer	Detection of *Aspergillus fumigatus* in a respiratory secretion	Fungal pathogens not detected	Fungal pathogens ot detected

*VV-ECMO*, veno-venous extracorporeal membrane oxygenation; *VA-ECMO*, veno-arterial extracorporeal membrane oxygenation

*Only small peripheral thrombi, already in organization

**These patients presented clinically with symptoms being compatible with HLH and showing 2–4/5 tested established guideline criteria for HLH, respectively

The cut-off of 5/8 positive criteria could thus have been reached in these patients, but the remaining three diagnostic criteria were neither tested during life time nor in retrospect

**Fig. 4:**
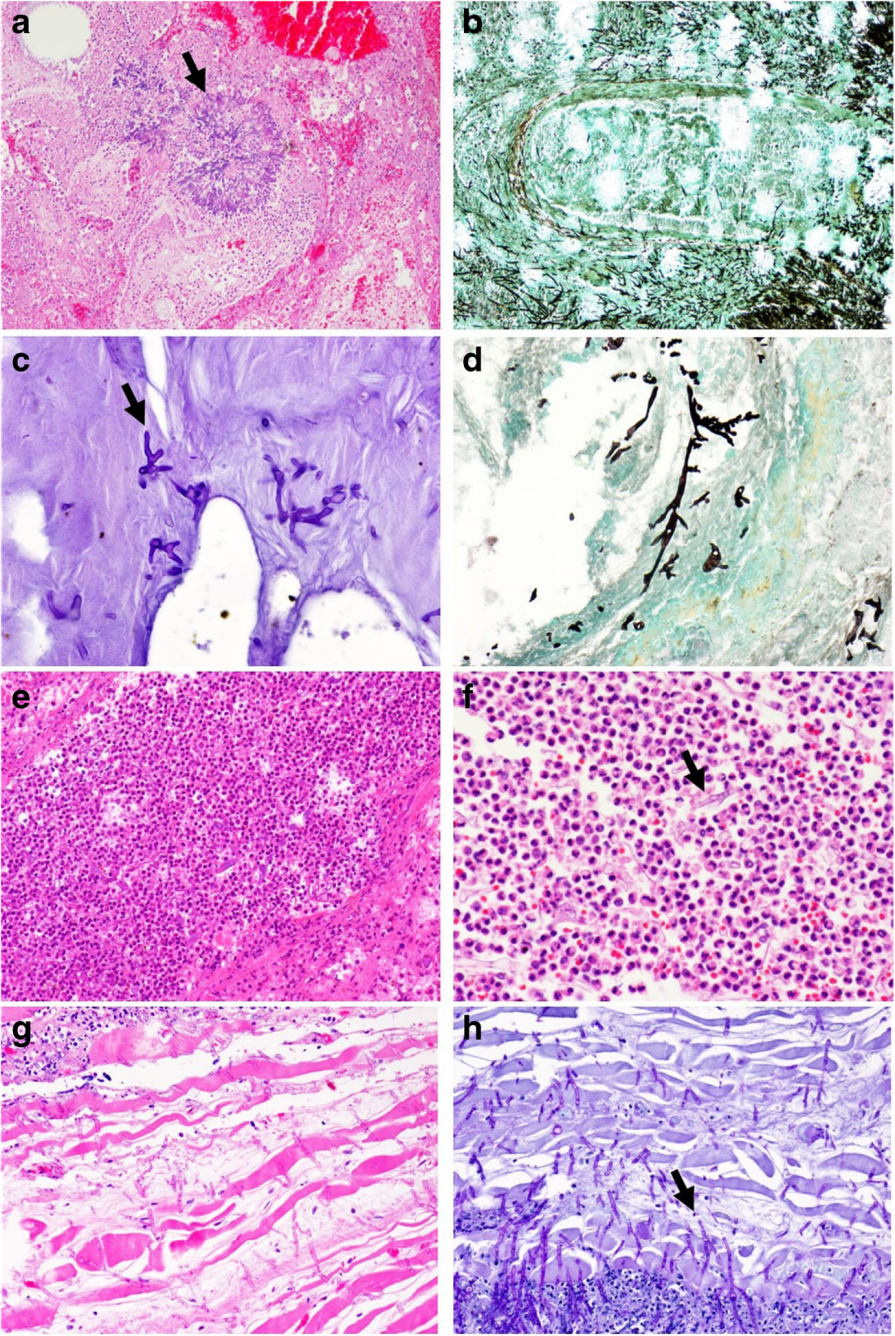
Fungal infection in different organs: Angionvasive aspergillosis of the lung (**a** P1, arrow, HE, original magnification × 100; **b** P1, Grocott, original magnification × 200). Zygomycetes species in the wall of the large bowel (**c** P3, arrow, PAS, original magnification × 400; **d** P3, Grocott, original magnification × 400). Acute inflammation of the lung with zygomycetes species (**e** P4, HE, original magnification × 200; **f** P4, arrow, HE, original magnification × 400). *Aspergillus* species within the wall of an esophageal ulcer (**g** P5, HE, original magnification × 200; **h** P5, arrow, PAS, original magnification × 200)

In addition to DAD, lung tissue was heterogeneously altered in a spectrum of more diffuse (Fig. 2c) or more often subpleural patchy tissue affection (Fig. 1a, 1b), suspicious of pneumonia. Indeed, as a new and the most important finding of this study, we observed particularly within these areas invasive mycosis with accompanying florid pneumonia (Fig. 4). In total, 50% of the patients showed invasive mycosis of the lung, aspergillosis in 3 cases, and mucor mycosis in one case. Interestingly, despite the clear-cut histological evidence, PCR analysis of two postmortal lung samples and one retrospectically analyzed antemortem respiratory secretion in P1 were similarly negative as one fungal culture of a respiratory secretion obtained antemortem and an *Aspergillus*-specific PCR analysis of the formalin-fixed, paraffin-embedded (FFPE) lung tissue specimen in P2, demonstrating the diagnostic power of postmortem histological analysis. Histopathology of autopsy specimens from P4 also revealed the presence of hyphae typical for mucormycetes in the lung. PCR analysis detected *Rhizopus microsporus/azygosporus* in tissue samples of the intestine, lung, and heart valve. Subsequently, antemortem patient samples were retrospectively subjected to PCR assessment, and 3/7 respiratory secretions as well as one EDTA stabilized blood sample contained nucleic acid of *Rhizopus microsporus/azygosporus*, indicating systemic invasive mucormycosis. However, all serum samples (*n* =8) were negative. FFPE tissue samples of the right lower lung lobe of P6 demonstrated regular shaped and septated mycelium suggestive for *Aspergillus* species, but PCR analysis of the corresponding FFPE sample again remained negative. However, one tracheal secretion collected antemortem and tested retrospectively provided evidence for *Aspergillus fumigatus*-specific nucleic acid. This finding could not be substantiated in further samples obtained from the respiratory tract (*n* = 6). On the opposite, a blood culture of P8 collected one day before the patient died revealed infection with *Candida albicans*, while histopathological investigations showed neither signs of fungal pneumonia or sepsis. PCR assays of 2 additional respiratory samples were also negative. All fungal cultures performed in multiple, antemortem collected, respiratory secretions of P1–P8 failed to grow molds.

#### Liver pathology

In 7 of 8 liver specimens, we found mild to moderate cholestasis (P2–P8), partly in the transition to sepsis-associated hepatic injury with marked cholestasis and single-cell necrosis (P2, P3, P5, P6, P7, P8) (Fig. 2d). Fatty degeneration of the liver occurred in 5 of 8 cases, with only two of them showing marked steatosis ranging from 30 to 50 % (P2, P3). Mild fibrosis was evident in two cases (P2, P6), while one liver showed complete cirrhosis of the liver (P4; Fig. 2e). This latter liver showed PAS-positive globules of alpha-1-antitrypsin in periportal hepatocytes, which proved to be type PiZ by immunohistochemistry (Fig. 2f).

#### Gastrointestinal tract

In a tissue biopsy of the large bowel wall obtained one day before P3 died, as well as in autopsy samples of the small and large bowel, irregularly shaped hyphae consistent with mucormycetes were detected (Fig. 4c, 4d). PCR confirmed this finding and discovered DNA specific for *Rhizopus microsporus/azygosporus* in the colon, gallbladder, and myocardium. All respiratory secretions (*n* = 7), serum samples (*n* = 8), and one lung biopsy were negative for fungal nucleic acid.

Fungal infiltration was also detected in an esophageal ulcer (P5) with regular-shaped hyphae compatible with *Aspergillus* species (Fig. 4g, 4h). The FFPE sample was subjected to PCR testing and nucleic acid specific for *Aspergillus fumigatus* and *Mucor* species were detected. No fungal nucleic acid was detected in serum (*n* = 15) and respiratory samples (*n* = 4).

#### Bone marrow changes

The bone marrow showed age-adjusted hypercellularity in all cases, mainly caused by reactive hyperplasia of the granulopoiesis. Additionally, marked siderosis was found in six cases (P1, P2, P3, P6, P7, P8). Signs of hemophagocytosis were evident in 7 of 8 cases (P1, P2, P4, P5, P6, P7, P8), with emperipolesis and PAS-positive macrophages (Fig. 2g) in the bone marrow, and highlighted by immunohistochemistry of CD68 (PG-M1) (DAKO, Code-Nr. M 0876) (Fig. 2h).

#### Skin

Histopathological examination of FFPE samples from P7 provided no evidence for invasive fungal disease, but yeast-like structures and inflammation without evidence for tissue invasion could be detected in a thoracic skin ulcer. Unfortunately, a reliable and more precise determination of the fungal structures was not possible and PCR testing remained negative.

#### Other organs

All other investigated organs (e.g., heart, spleen, kidney, thyroid, salivary and adrenal glands, pancreas) showed no relevant pathological changes other than secondarily related to (septic) multi-organ failure.

#### Cause of death

The cause of death was a multi-organ failure in all cases. In 6 cases, this was due to sepsis, and four of these were attributed to invasive mycosis. In two other cases, multi-organ failure developed secondarily to primary severe respiratory failure. In conclusion, patients’ death could be attributed to fungal sepsis in 50% of the cases in this series.

### Electron microscopical findings

Ultrastructural analyses of representative organs were performed for the potential detection of virus-like particles in different compartments. In 3 of 8 (P2, P3, P8) cases, we found repetitive round-shaped electron-dense particles with an average diameter of about 90 nm containing spike-like structures in alveolar cells and vesicles of respiratory cells of the lung (Supplementary Figure 1). However, severe autolytic changes prevented a clear-cut evidence of virus particles in all cases.

### SARS-CoV-2 PCR and isolation

To gain more insight into the presence of SARS-CoV-2 in different organs, we analyzed freshly frozen organ slices using a quantitative PCR. As shown in Table 1, we detected SARS-CoV-2 RNA up to a concentration of 7 × 10^5^ copies/100.000 cells in the lung (*n* = 6), liver (*n* = 3), kidney (*n* = 3), trachea (*n* = 2), heart (*n* = 2), and gut (*n* = 1), thus providing evidence of a persistent systemic infection in 7 of 8 patients after 16–56 days of medical treatment.

## Discussion

The main goal of this study was to examine the spectrum of autopsy findings in COVID-19 patients after long-term medical treatment, with a main focus on microbiological findings and particular emphasis on mycotic infections. Compared with an average autopsy rate of less than 4% in Germany [13], we reached an autopsy frequency of 47% in COVID-19 patients. This was obviously mainly the direct consequence of the bigger interest of the clinicians to perform autopsies; but it could also—at least partly—be attributed to the high willingness of relatives to agreement for performing autopsies to gain additional knowledge of this novel disease. Infections among the autopsy staff did not occur, and all performed antigen tests were negative, given the implementation of special safety precautions. All deceased patients suffered from non-severe comorbidities like hypertension and obesity. One severe previously known disease was liver cirrhosis in one patient (P4). This was clinically attributed to persistent alcohol abuse. Autoptically, however, we detected alpha-1-antitrypsin deficiency in this patient, whose course presumably was unfavorably influenced by the elevated alcohol consumption. This diagnosis alone underlines the general importance of autopsies, as this diagnosis will naturally have impact on the relatives.

Compared with other autopsy studies (mean: 72–79.2 years old [2, 8, 11, 14]), our patients were younger, with a median age of 62 years. In addition, they showed a longer hospitalization time (median 33.6 days) compared to other autopsy studies (6–16 days) [2, 8, 11, 14]. The younger age of our patients is presumably a consequence of the strict ECMO-criteria at the University Hospital of Regensburg. As a result of long-term maximum therapy (including mechanical ventilation partly combined with a prone position, dialysis, ECMO, and convalescent plasma) and the younger age, patients survived for a longer time. This gave us the opportunity to investigate a group of long-term treated patients with possibly a different spectrum of diseases and findings that have not yet been systemically investigated so far. In line with the hypothesis of time-related changes of alterations in autopsy findings, we found a relatively low rate and generally minor manifestation of the pathologic alterations that have been described as being typical for the early phase of the disease, such as endothelitis, thrombosis, or capillaritis [2]. However, as a result of the pulmonary infection, we noticed severe damage and fibrosis, and remodeling of the lung parenchyma corresponding to different stages of DAD in 7 of 8 cases. Corresponding to the long-term ventilation, most of the patients showed DAD already in early fibrotic stage. Only one patient showed a severe pulmonary fibrosis with dens collagene deposits (Fig. 3e), leading to death in respiratory failure. Virus-infected cells similar to those observed in the course of cytomegalovirus or adenovirus infection with virus inclusions were not detected.

Importantly, structures that may represent autolytic virus particles were identified only in 3 of 35 electron microscopy samples. Actually, the potential of identifying coronavirus by electron microscopy remains a matter of debate [19], particularly in autopsy samples. The fact that in most of the analyzed specimens no or at best a small number of “suspicious” structures was found underlines the insensitivity and the lack of specifity of electron microscopy for this scope, at least in the post mortem context. Therefore, confirmatory immune electron microscopic examinations, which, however, are still pending, are mandatory to better understand the infectious route of the virus in different human tissues. Currently, molecular methods such as PCR seem to be better suited for virus detection in tissues, although they cannot locate the virus in a cell-specific manner.

PCR analyses of viral nucleid acids showed positive results in different organs (lung 6/8, trachea 2/3, heart 2/8, liver 3/8, kidney 3/8, heart 2/6, gut 1/1) in this study, with the highest detectable virus copy numbers in the lung (maximum 7*10^5^ cop./ml). To further investigate whether the virus is still fully capable of replication requires the isolation and cultivation of SARS-CoV-2 via cell culture in future studies.

The clinically most important finding of this examination was the detection of invasive mycosis in inner organs in all but two cases. Moreover, 50% of the patients died of the consequences of mycotic sepsis. In general, aspergillus infections occur on average in about 10–12% of patients with DAD of any reason [6, 18]. Of note, specific mycosis was never diagnosed in our cohort clinically or microbiologically antemortem, despite prophylactic calculated antimycotic treatment (caspofungin or isavuconazole), which, on average, was given for 9.5 days. In addition, despite clear-cut histopathological evidence of angioinvasive pulmonary mycosis, subsequent molecular analyses were unable to detect fungal DNA in related FFPE samples. The lack of microbiological detection of fungi in these samples might be the known consequence of formalin fixation and the resulting DNA degradation [3], which hampers the results of microbiological methods that are designed to analyze fresh samples.

Particularly interesting was the finding of invasive infections with mucormycetes in P3 and P4. PCR testing of various samples obtained antemortem and postmortem were positive for *Rhizopus microsporus*/*azygosporus* DNA, indicating systemic mucormycosis originating from the gastrointestinal (P3) and respiratory tract (P4), respectively. *Rhizopus* DNA could also be detected in a retrospectively analyzed EDTA blood sample of P4, but all serum samples were negative. However, other studies reported better results when testing serum samples of patients at risk of mucormycosis [20]. Patients with severe, community-acquired viral pneumonia, i.e., influenza, or receiving long-term ventilation are as yet not known to be at risk of superinfections with mucorales [5]. Mucormycosis is typically a rare infection described in severely immunocompromised patients, suffering from hematological malignancies, solid tumors, ketoacidosis due to diabetes mellitus, transplantation, treatment with steroids and myelosuppressive agents, iron chelator therapy, and traumatic skin and soft tissue infections [22]. P3 and P4 received hydrocortisone treatment, equivalent to 60 mg prednisolone/day, which is in the range of the recommended Dexamethasone dose for severe COVID 19 patients, before the first evidence of fungal infection was detected. However, this does not exclude that the infection preceded the administration of steroids. Steroids might have been a co-factor in the development of mucormycosis, although in general this is not a very frequent complication of steroid therapy. Additional factors may have contributed. For instance, P3 did not produce SARS-CoV2 antibodies during the disease. Therefore, an unknown immunodeficiency might be present in this patient. Furthermore, the immune modulation described in severe COVID-19 [21] might specifically support the development of mucormycosis in long-term ventilated SARS-CoV-2 patients. There is one additional study describing superinfections of COVID-19 patients with mucormycetes. The retrospective study included 257 patients in the Chinese province of Jiangsu from January 22 to February 2 and reported infections with *Mucor* spp. in 6 cases [27]. However, these patients suffered from mild to moderate disease and there was no histologic confirmation. In addition, one other autopsy study showed mucor infection in 1/10 investigated cases. This patient was intubated for 22 days but other clinical information could not be obtained from this report. Nevertheless, this shows that mycotic and specifically mucor infections are not a local phenomenon and may particular gain importance during the course of the disease [11].

Histopathology of two patients (P5, P7) revealed locally restricted fungal infections, which might be superinfections due to previous damage to the mucosal or skin barrier. In P5, PCR found molds (*Aspergillus fumigatus* and *Mucor* species) consistent with the microscopic detection of hyphae. Yeast-like elements in the skin sample of P7 could not be specified with fungal PCRs; however, the patient was colonized with *Candida parapsilosis* in the respiratory and urogenital tract. *Candida parapsilosis* is prone to colonize the human skin [25]; therefore, this yeast might have superinfected the preexisting skin ulcer.

Histopathological and mycological findings in P1, P2, and P6 indicated invasive pulmonary aspergillosis, which is an infectious complication in critically ill patients and known as superinfection in ventilated influenza patients [15, 26], and has already been described in patients with severe SARS-CoV-2 pneumonia [24].

P8 had fungal bloodstream infection with *Candida albicans* grown in blood cultures obtained 1 day before the patient died. Candidemia is a well-known nosocomial infection in intensive care patients, [25] often due to intravascular catheters infection or damaged mucous membranes of the gastrointestinal tract.

Systemic fungal infections in COVID-19 patients have been described in individual cases [11, 24], but we were able to detect them in 6 of 8 investigated patients in this study. In line with Gangneux [9], we recommend considering invasive mycosis including mucormycosis as a complication in long-term ventilated SARS-CoV-2 patients.

Additionally, we could find signs of a hemophagocytic lymphohistiocytosis (HLH) with increased phagocytosis of bone marrow cells by macrophages in bone marrow specimens of all but one patient (Table 1), similar to the findings of Hanley et al. [8]. In P6 and P7, this finding was in line with the clinical presentation as HLH was already evident at life time (fulfillment of 5/6 tested criteria for HLH, according to the HLH-2004 guidelines [12]). In P3, P4, and P5, the clinical presentation was not suspicious for HLH, while in P1, P2, and P8, HLH was possible (3/5, 2/5, and 4/5 positive HLH-2004-guideline criteria). The latter group was designated as “maybe HLH” in Table 1, because additional testing of the remaining three criteria could have allowed for fulfillment of the criteria and the clinical presentation was compatible with HLH. The higher incidence of macrophage activation syndrome in patients with COVID-19-infection has already been described [5, 8, 18]. It is regarded as being a consequence of the hyperinflammatory state [18] of the patients which is confirmed by our study as 7/8 patients showed hyperinflammation according to the IL-6 levels (Table 1).

Conclusively, the results of this study underline the importance of performing autopsies, especially in gaining better insights into unknown or poorly defined diseases like SARS-COV2. Therefore, the close interaction between the clinicians and the pathologists as well as microbiologists is essential for the accurate assessment of the findings obtained during the autopsy and the possible resulting adjustment of diagnostics and therapy in the future. All our patients had comorbidities, but only one patient was ill enough that she could possibly have died even without the SARS-CoV-2 infection as a result chronic liver failure with acute liver decompensation in the background of liver cirrhosis. The findings of macrophage activation syndrome together with fungal superinfections suggest that long-term treated COVID-19 patients suffer from severe immunopathologies that favor fungal (super)-infections, particularly *Mucor* and *Aspergillus* species. Finally, we have demonstrated that clinically undetected fungal infections are a major cause of death in COVID-19 patients after long-term treatment. We believe that this important finding warrants alterations in current microbiological screening strategies that should also include the implementation of suitable diagnostic methods already in local hospitals, to allow for an early diagnosis and subsequent specific and thus hopefully successful antifungal treatment.

## Supplementary Information

ESM 1 (DOCX 300 kb)

## Data Availability

All data and material are available.
