# Supplementary material for: Autopsy findings after long-term treatment of COVID-19 patients with microbiological correlation
Source: Virchows Arch. 2021 Jan 20;479(1):97–108. doi: 10.1007/s00428-020-03014-0 (PMC7816067; doi:10.1007/s00428-020-03014-0)
Supplement: Supplementary file 1 — (DOCX 300 kb) [file 428_2020_3014_MOESM1_ESM.docx]

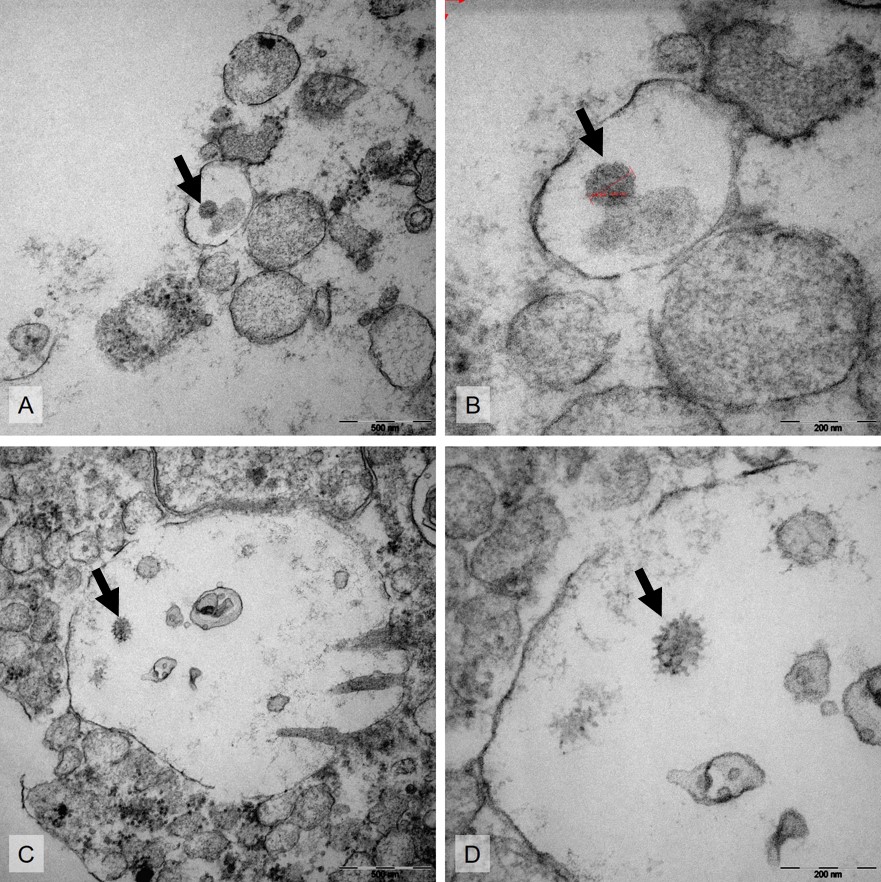


**Supplementary Figure 1: Electron microscopical findings:** Autopsy samples of electron microscopical anlayses showed severe signs of autolyses. At best, "virus-like" particles could be detected by transmissionelectron microscopical analyses in alveolar cells (A-D; P2, P3, arrow) with spike-like structures (B, P2; D, P3), while the nucleocapsid and the typical crown-like spikes of SARS-CoV2 have not been recognized at all.

| **Patient characteristics at the UKR Intensive Care Unit** | |
| --- | --- |
| COVID-positive patients with intensive care therapy | 55 |
| COVID-positive patients who died at the UKR | 17 (30,9 %) |
| **Autopsies of COVID-positive deceased UKR** | 8 (47,1 %) |
| Male/Female | 4/4 |
| Median Age | 62 (44 - 73) y |
| Body Mass Index | 30,2 (20,8 - 40,4) kg/m2 |
| Days beeing hospitalized | 33,6 (16 - 56) |
| Days on mechanical ventilation | 29,6 (13 - 51) |
| Days between onset of first symptoms and death | 35,3 (21 - 54) |
| **Therapy** |  |
| ECMO | 5/8 (1 x VV-ECMO, 2 x VA-ECMO, 2 x VV- and VA-ECMO) |
| Dialysis | 8/8 |
| Days on Dialysis | 19,5 (1 - 49) |
| Days on Antibiotics | 29,6 (2 - 56) |
| Days on Antimycotics | 9,5 (0 - 36) |
| Preexisting immunsuppressive Therapy | 0/8 |
| Immunsuppressive Therapy | 8/8 (7 Anakinra, 1 Tozilizumab) |
| Prone Position Ventilation | 8/8 |
| Convalescent plasmaTreatment | 4/8 |
| **Hyperinflammatory state**  IL-6 | 7/8  3702,6 pg/ml (1728 - 9296pg/ml) |
| **Comorbidities**  Hypertension  Obesity  Liver disease (NASH, liver cirrhosis, etc.)  Diabetes  Others* | 5/8  6/8  2/8  1/8  8/8 |
| **Clinical causes of death**  Multi organ failure  Sepsis  Liver failure | 8/8  4/8  1/8 |

**Supplementary Table 1:** **Patient characteristics including information on therapy and comorbidities** (VV-ECMO: veno-venous extracorporeal membrane oxygenation; VA-ECMO: veno-arterial extracorporeal membrane oxygenation; * Depression (1/8), COPD (1/8), Diabetes Mellitus (1/8), Abdominal Surgery (1/8), Sleep Apnea Syndrome (1/8), Factor V Leiden Deficiency (1/8), Atrial Fibrillation (2/8))
